# Safety and Efficacy of Subcutaneous Buttock Augmentation in Indian Population: A Retrospective Analysis

**DOI:** 10.1055/s-0045-1809330

**Published:** 2025-06-02

**Authors:** Rajat Gupta, Priya Bansal, Gautam Chaudhury, Nandini Singh Tanwar

**Affiliations:** 1 Department of Plastic Surgery, Excel Hospital, C K Birla Hospital, and Rosewalk Hospital, New Delhi, India; 2 Department of Plastic and Reconstructive Surgery, Excel Hospital, CK Birla Hospital, and Rosewalk Hospital, New Delhi, India; 3 Department of Burns, Plastic and Reconstructive Surgery, All India Institute of Medical Sciences, New Delhi, India

**Keywords:** safe subcutaneous buttock augmentation, Brazilian butt lift, gluteal anatomy, fat grafting, expansion vibration lipofilling, waist-to-hip ratio, cosmetic surgery

## Abstract

**Background:**

Safe subcutaneous buttock augmentation (SSBA) offers a safer alternative to traditional gluteal fat grafting, addressing concerns of complications such as fat embolism. This study aims to establish the safety and efficacy of SSBA in an Indian cohort.

**Materials and Methods:**

A retrospective analysis was conducted on 293 patients (287 females, 6 males) who underwent SSBA between January 2017 and September 2024. Fat was harvested using ultrasound-assisted liposuction and power-assisted liposuction, followed by grafting into the subcutaneous plane using a power-assisted device with a 5-mm blunt cannula.

**Results:**

The mean fat grafting volume for females was 557 mL and 341.6 mL for males. There were no reported mortalities, and complications were minimal. For females, the mean waist-to-hip ratio improved significantly from 0.81 to 0.72 ( *p* < 0.001), demonstrating notable aesthetic enhancement.

**Conclusion:**

SSBA is a safe and effective procedure for buttock enhancement, showing significant improvement in body contour and minimal risk of complications. These results reinforce the importance of technique precision and safety protocol adherence.

## Introduction

Due to the high mortality rate of Brazilian butt lift (BBL) at 1:3,000, 1 2 3 4 5 BBL gained notoriety as one of the most dangerous cosmetic procedures, prompting some countries to advise against its practice. Recent epidemiological studies have further highlighted these safety concerns, emphasizing the need for modified techniques. 6 In response, the aesthetic surgery community formed a Task Force to develop safer guidelines and redefined the procedure as safe subcutaneous buttock augmentation (SSBA). 7 8

Buttock augmentation has gained significant popularity worldwide, including in India, driven by increasing aesthetic awareness and advancements in surgical techniques. SSBA prioritizes patient safety by confining fat graft placement strictly to the subcutaneous layer, thereby significantly reducing the risk of fat embolism—a critical concern associated with traditional BBL techniques. By adhering to these enhanced safety protocols, SSBA has emerged as a safer and more controlled approach to buttock augmentation. This advancement has been particularly beneficial for clinics serving both domestic and international patients seeking safe and effective cosmetic procedures.

This article demonstrates the suitability and success of SSBA in the Indian population with different needs and goals. It underscores the importance of adopting SSBA as the standard for modern aesthetic practice for patients desiring buttock augmentation.

## Materials and Methods

This study employed a retrospective design, with postoperative assessments performed over a period of 6 months to 1 year to evaluate patient outcomes and procedural effectiveness. Between January 2017 and January 2024, a total of 293 SSBA procedures were conducted, including six male patients. The study was approved by the GeneBandhu ethics committee (Ref- ECG035/2024). The meeting was held on December 7, 2024.

### Preoperative Considerations

A thorough preoperative assessment was conducted for all patients, including blood investigations and evaluation of venous thromboembolism (VTE) risk factors such as prior VTE, prolonged bed rest, use of estrogen or herbal medications, cancer, thrombophilia, and family history of VTE. Lower limb examination, electrocardiogram, and chest X-rays were performed. Anesthetist clearance was mandatory, and patients were advised to stop smoking, drinking green tea, and consuming supplements at least 1 week before surgery.

**Fig. 1:**
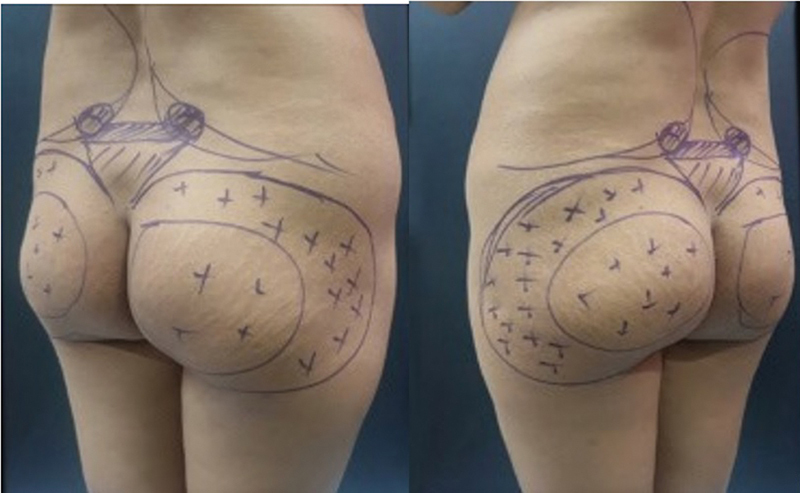
Preoperative Markings.

On the day of surgery, donor and recipient areas were marked ( Fig. 1 ). Buttock contour was marked as an outline and areas of fat injection were marked with (+) sign. Waist and hip circumferences were measured, and access incisions for liposuction and lipofilling were preplanned. Markings also depended upon the areas and extent of result patients wanted. Detailed discussion with patient is done with respect to their desired shape. Three areas are discussed, namely, (1) enhancing buttock projection—central part of buttocks need augmentation and are marked, (2) correction of hip dips—hip dip zones are marked, and (3) width enhancement—lateral buttocks are marked. Many patients especially females want a combination of the above three areas. In contrast, however, male patients usually ask for enhancing projection of buttocks only. Standardized photographs were taken, and patient preferences for buttock shape were documented.

### Intraoperative Procedure ( Video 1 )



**Video 1** Video explaining the intra-operative procedure of Safe Subcutaneous Buttock Augmentation (SSBA).


SSBA was performed under general anesthesia with deep vein thrombosis pumps applied. Foley catheterization was performed, and sterile preparation was extended from the nipple line to the infrapatellar region. Access incisions were strategically placed at specific anatomical sites for liposuction.

Preoperative skin markings were performed on the day of surgery. Following the induction of anesthesia as per protocol, tumescent infiltration was administered using a solution comprising 1 L of normal saline, 10 mL of 2% lignocaine, one ampoule of adrenaline, and one ampoule of tranexamic acid. The solution was evenly distributed using a basket cannula attached to the power-assisted liposuction (PAL) system. The infiltration volume was predetermined based on the amount of fat to be aspirated, maintaining a ratio of 1:1 to 1:2.

The SAFELipo technique (Separation, Aspiration, and Fat Equalization) was employed, utilizing VASER (Vibration Amplification of Sound Energy at Resonance) and PAL devices. Fat emulsification was performed with a VASER probe at 70% power in pulsed mode, followed by aspiration using power-assisted cannulas at 500 mm Hg.

Patients were then positioned prone (jackknife position) with sterile preparation extending from the scapula to the mid-thigh. Back liposuction, following the same SAFELipo method, focused on the sacral V and lower back in female patients to enhance buttock projection. In male patients, less aggressive liposuction was performed in lower back to maintain a masculine silhouette. Fat injected was also limited to a volume that corrected/enhanced the existing deformity/proportions, most commonly buttock projection rather than exaggerating the lateral curves. The goal was to achieve a balanced and proportionate result that complemented the patient's overall body contour.

Liposuction access incisions were closed using Monocryl 3–0 sutures.

### Fat Processing and Grafting

**Fig. 2:**
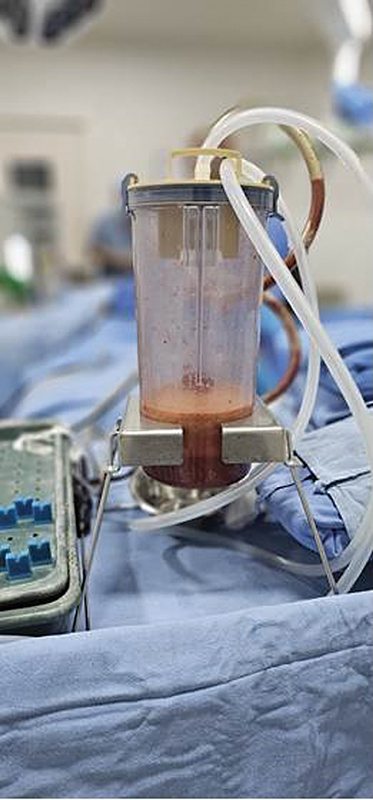
Harvested fat collected in Sterile Jar.

**Fig. 3:**
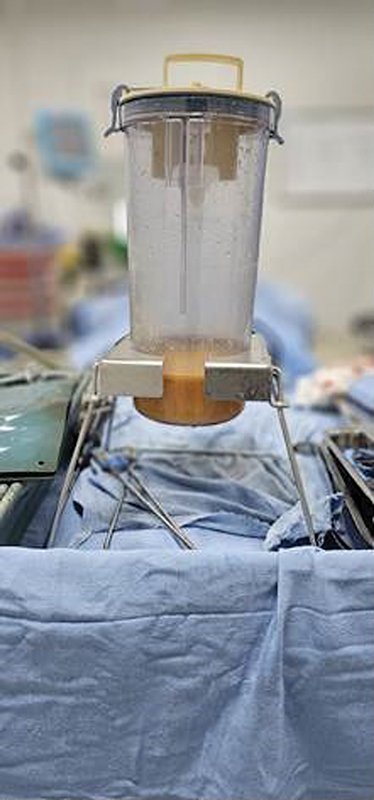
Harvested fat washed, sedimented and purified.

**Fig. 4:**
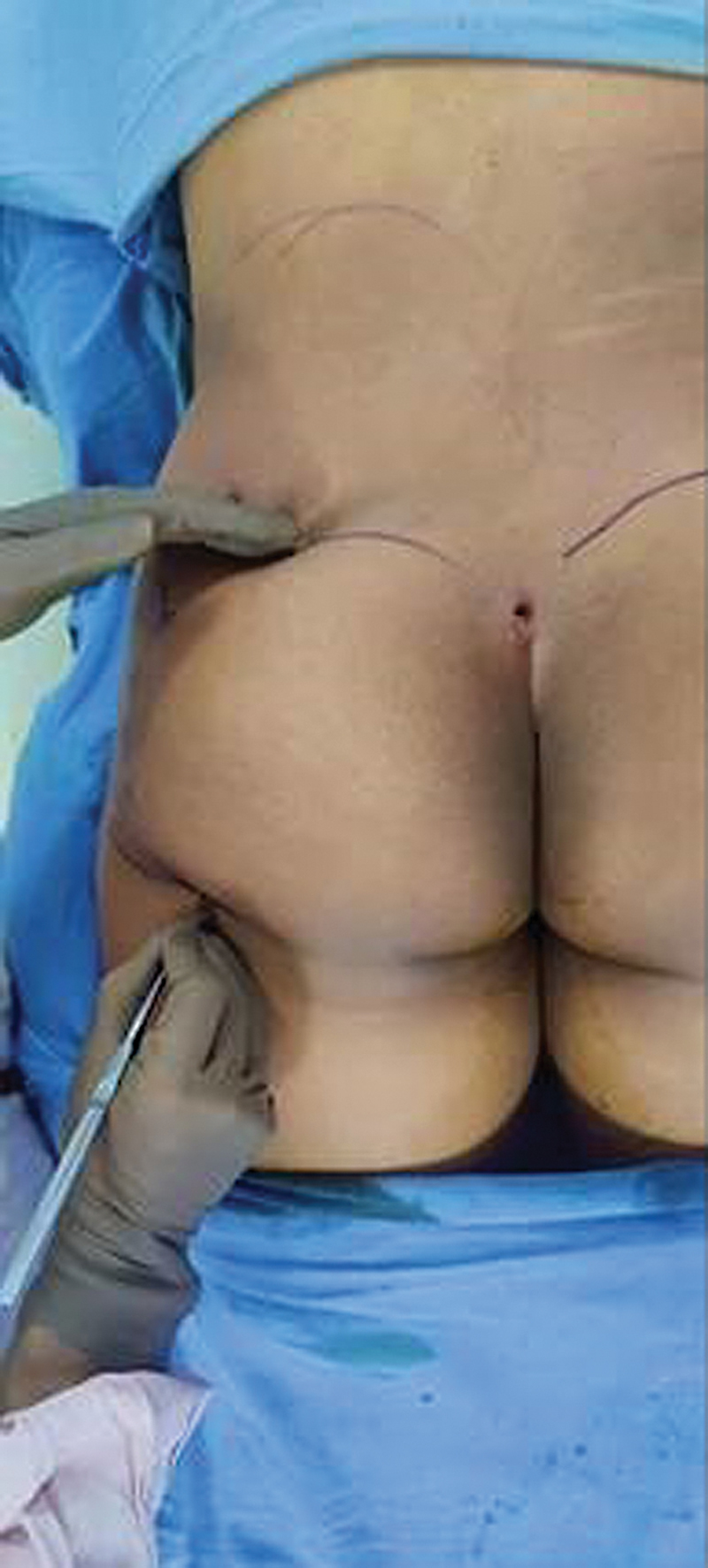
Site of stealth incisions in buttock cleft and buttock crease.

**Fig. 5:**
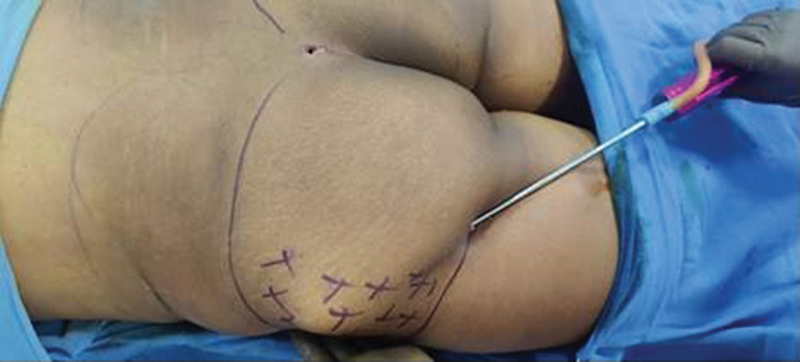
EVL technique in progress with canula in subcutaneous plane.

The aspirated fat was washed, sedimented, and purified ( Figs. 2 and 3 ). Stealth incisions were made just above the buttock cleft and in the butt crease on each side ( Fig. 4 ). A subcutaneous-only strategy was used for fat grafting, employing the expansion vibration lipofilling (EVL) technique ( Fig. 4 ). A blunt 5-mm basket angled cannula of 40 cm length ( Fig. 5 ) was attached to a power-assisted device, and fat was grafted to the buttocks, hip dips, and marked recipient areas. The gluteal danger triangle was carefully avoided. Grafting continued until skin tautness was achieved, and overfilling was avoided to promote fat survival. For male patients, smaller graft volumes were used to maintain masculine proportions.

### Postoperative Care ( Fig. 6 )

Patients were placed in a prone position to avoid buttock pressure. Vital parameters were monitored, and chemoprophylaxis with enoxaparin (40 mg/day) was initiated for high-risk cases. Pain management, early ambulation, and fluid balance were priorities. Discharge was same day for graft volumes up to 450 mL or less than 2.5 L liposuction; larger volumes required overnight observation. Patients received oral antibiotics (cefixime 200 mg twice daily for 5 days) and compression garments for donor sites, which were worn for 6 weeks. Prone positioning was maintained for 3 weeks, with sitting allowed only on BBL pillows. For patients who underwent simultaneous abdominoplasty, off-loading was achieved by placing pillows below the lower back and upper thighs. Massaging of donor sites was permitted after 2 days, but massaging the buttocks was restricted. Follow-ups occurred on postoperative days 3, 7, and 21, with ongoing patient support provided via phone or video for 6 months ( Figs. 7 and 8 ).

**Fig. 6:**
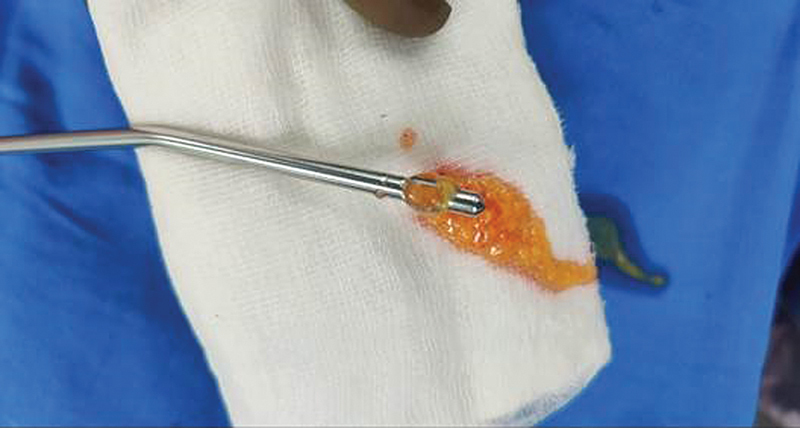
5 mm basket power assisted cannula used to graft fat connected through pressure pump.

**Fig. 7:**
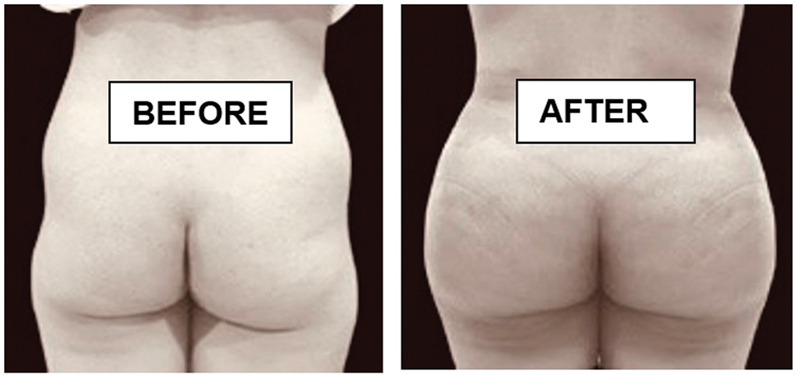
6 months post op of 450 cc of fat grafted on each side for this 27-year-old female.

**Fig. 8:**
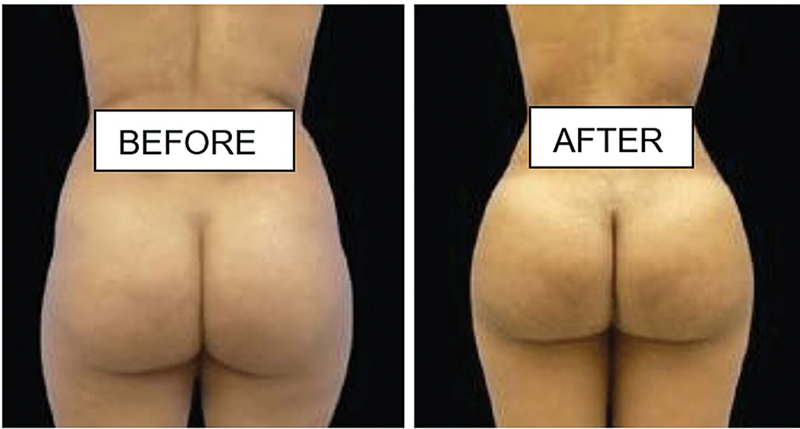
6 months post op of 600 cc of fat grafted on each side for this 35-year-old female.

Comprehensive clinical data were collected, encompassing patient demographics, fat graft volumes, pre- and postoperative waist-to-hip ratios, complications, and additional procedures. Statistical analysis included subject profiling by gender and age. Changes in waist-to-hip ratios were evaluated using a paired *t* -test, with a significance threshold of *p* < 0.05. All analyses were conducted using SPSS software, ensuring accuracy and reproducibility of results.

## Results

From January 2017 to January 2024, 293 patients underwent SSBA procedures. The patients were followed up for a minimum of 6 months and a maximum of 1 year. Out of the 293 patients, 6 were males and the rest 287 females.

The mean age of male patients was 28.3 years (range 23–36 years) ( Table 1 ). The mean amount of fat grafted was 341.6 mL (range 250–400 mL) ( Table 2 ). The preoperative waist:hip ratio ranged from 0.76 to 0.88 (mean = 0.82), while the postoperative waist:hip ratio ranged from 0.74 to 0.85 (mean = 0.8) ( Table 3 ).

**Table 1:** Distribution of male patients who underwent SSBA by age group

Age group	Number ( *n* = 6)	Percentage
20–25	3	50.0
26–30	1	16.7
31–35	0	0.0
36–40	2	33.3

Abbreviation: SSBA, safe subcutaneous buttock augmentation.

**Table 2:** Amount of fat grafted in male patients who underwent SSBA (in mL): descriptive statistics

Parameter ( *n* = 6)	Value
Mean	341.6
SD	66.45
Range	250–400

Abbreviations: SD, standard deviation; SSBA, safe subcutaneous buttock augmentation.

**Table 3:** Change in waist:hip ratio of male patients ( *n* = 6) by SSBA

	Mean	SD (range)
Preoperative	0.82	0.047 (0.88–0.76)
Postoperative	0.8	0.047 (0.85–0.74)
Difference	0.02	0.01 (0.04–0.01)

Abbreviations: SD, standard deviation; SSBA, safe subcutaneous buttock augmentation.

Female patients ranged from 24 to 40 years of age with the mean age being 30.76 years. A little less than three-fourths (73.5%) were in the age group 26 to 35 years ( Table 4 ). The amount of fat grafted into individual buttock ranged from 350 to 800 mL with the average being 557 mL per buttock ( Table 5 ).

**Table 4:** Distribution of female patients who underwent SSBA by age group

Age group	Number ( *n* = 287)	Percentage
20–25	43	15.0
26–30	82	28.6
31–35	129	44.9
36–40	33	11.5

Abbreviation: SSBA, safe subcutaneous buttock augmentation.

**Table 5:** Amount of fat grafted in female patients who underwent SSBA (in mL): descriptive statistics

Parameter value ( *n* = 287)	Value
Mean	557
Range	300–800
Standard deviation	121.7
Coefficient of variation	21.84%

Abbreviation: SSBA, safe subcutaneous buttock augmentation.

**Fig. 9:**
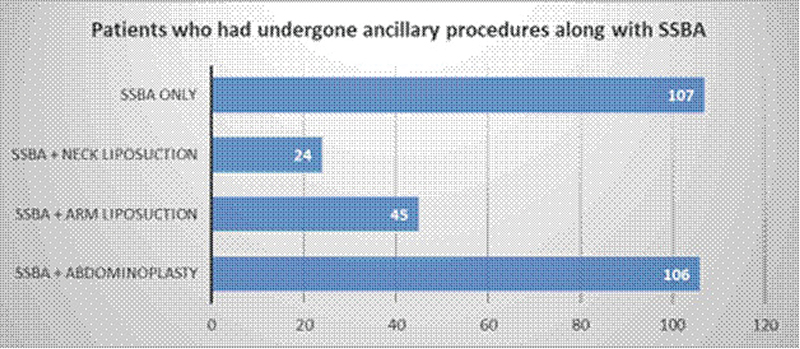
Distribution of Ancillary Procedures Performed Alongside SSBA.

Of 287 female patients, 107 had only SSBA. Further, in addition to SSBA, 106 patients had abdominoplasty, 45 patients had arm liposuction, and 24 patients had neck liposuction ( Fig. 9 ).

In a cohort of 293 patients who underwent SSBA, no cases of mortality, seroma, cellulitis, or sciatic nerve complications were reported. Postoperative complications were minimal, primarily consisting of mild bruising at the donor sites and slight buttock irregularities, which resolved with gentle massages to the area.

One patient who underwent concurrent abdominoplasty experienced minor wound dehiscence, which was successfully treated with secondary suturing. Another patient required a revision SSBA due to significant fat resorption (65–70%), which was attributed to noncompliance with postoperative care; this was rectified following improved adherence to the prescribed postoperative guidelines. Additionally, one patient voluntarily sought a second SSBA to enhance the aesthetic outcome.

Preoperative waist-to-hip ratios averaged 0.79 (range 0.76–0.86), while postoperative ratios averaged 0.72 (range 0.69–0.83), showing a statistically significant reduction of 0.07 (standard deviation: 0.025), with a paired *t* -test value of 27.8 ( *p* < 0.001) ( Table 6 ).

**Table 6:** Change in waist:hip ratio of female patients ( *n* = 287) by SSBA

	Mean	SD (range)
Preoperative	0.79	0.019 (0.76–0.84)
Postoperative	0.72	0.017 (0.69–0.75)
Difference	0.07	0.024 (0.02–0.13)

Abbreviations: SD, standard deviation; SSBA, safe subcutaneous buttock augmentation.

Note: Value of paired *t* -test = 27.8, *p* < 0.001 (highly significant).

## Discussion

The growing demand for fuller buttocks has led to a surge in buttock augmentation procedures. This trend has been amplified by high-profile celebrities who have popularized curvier body types. While buttock implants were once the go-to method for augmentation, concerns over complications such as displacement, infection, and capsular contracture have shifted preferences toward buttock fat grafting. This technique, commonly known as the BBL, 9 involves large-volume lipofilling into the buttocks and has seen a global rise of over 200% in surgeries. However, despite its perceived safety, BBL has been marred by significant risks, particularly fat embolism. The most severe complication of fat embolism in this procedure is pulmonary fat embolism due to injury to adipose tissue & small blood vessels which release fat particles into venous system causing pulmonary injury. Symptoms like altered mental status, neurological deficits or skin rash should raise suspicion of pulmonary fat embolism, warranting prompt diagnostic evaluation. 10

This has resulted in fatalities highlighting the need for improved safety protocols. One such safety review & recommendations is proposed by British Association of Aesthetic Plastic Surgeons (BAAPS). 11 The most severe complication of fat embolism in this procedure is pulmonary fat embolism due to injury to adipose tissue & small blood vessels which release fat particles into venous system causing pulmonary injury. Symptoms like altered mental status, neurological deficits or skin rash should raise suspicion of pulmonary fat embolism, warranting prompt diagnostic evaluation. 10

This has resulted in fatalities highlighting the need for improved safety protocols. One such safety review & recommendations is proposed by British Association of Aesthetic Plastic Surgeons (BAAPS). 11

Critics argue that “BBL” is a misnomer, as the procedure was not first performed in Brazil and does not involve skin resection or gluteopexy, calling it a lift! 7

The correct terminology should be SSBA, as fat is grafted specifically in the subcutaneous fat layer rather than the intramuscular fat deposits, which are believed to be the primary cause of fat embolism in BBL procedures.

The cause of massive intraoperative fat embolism in BBL surgeries has been widely debated, with the Siphon Theory, proposed by Del Vecchio, 12 being the most accepted explanation. This theory suggests that fat breaches the walls of deep gluteal veins and passively migrates following intramuscular fat deposition. Postmortem findings of BBL fatalities support this theory, as all deceased patients had intramuscular fat deposition, whereas no patient with only subcutaneous fat grafting succumbed to fat embolism. 2 13 This underscores the importance of ensuring fat is only deposited in the subcutaneous layer, avoiding the intramuscular plane to reduce the risk of complications.

The introduction of Coleman's technique 14 revolutionized fat grafting, using a small-caliber cannula and a syringe withdrawal technique to inject centrifuged fat. While this method is effective for most body areas, it yields suboptimal results in the gluteal region due to the presence of multiple fibrous septations, which create a tight compartment with limited capacity for expansion. Excess fat deposition can lead to increased interstitial pressure, causing fat necrosis. Two key disadvantages of Coleman's technique in buttock augmentation include the risk of “flexibility misguidance,” 1 where the cannula may bend, causing the surgeon to mistakenly graft fat intramuscularly, and upper extremity fatigue from syringe lipofilling, which can impair proprioception and lead to errors in cannula placement.

In contrast, the EVL technique addresses these limitations by employing a large-caliber cannula with a basket tip attached to a PAL device ( Table 7 ). This method creates a potential space in the gluteal subcutaneous layer through oscillations, allowing for more predictable and stable fat grafting. The EVL technique facilitates simultaneous fat grafting and tissue expansion, improving fat survival by enhancing vascularity and graft homogeneity. These advantages make EVL a more effective and reliable method for SSBA compared with the Coleman's technique. 1 2 15

**Table 7:** Comparison of Coleman's technique versus expansion vibration lipofilling (EVL) technique

Sl. no.	Coleman's technique of fat grafting	Expansion vibration lipofilling technique
1.	Fat separated by centrifugation	Fat separated by sedimentation
2.	Small caliber cannula may lead to “flexibility misguidance” and accidental intramuscular fat grafting	Large caliber cannula with basket tip reduces accidental intramuscular fat grafting
3.	Fat is grafted into available space	Potential space is created through oscillations
4.	Fat is injected using a syringe withdrawal technique	Fat is propelled by a pump during lipofilling
5.	Leads to more hand muscle fatigue, altering proprioception	Less hand fatigue, better proprioception, enhancing cannula tip placement accuracy
6.	Limited fat grafting in buttocks	Greater fat volume can be grafted in the subcutaneous layer
7.	Unpredictable fat survival in the gluteal region	Improved fat survival, leading to more predictable outcomes

Furthermore, the procedure is not solely focused on buttock enhancement; the majority of body contouring comes from liposuction of the lower back and waist, with lipofilling serving as a secondary procedure to enhance gluteal projection.

SSBA should be seen not just as a procedure to enlarge the buttocks but as a comprehensive body contouring method. Aggressive liposuction of the sacral V and flanks is essential, 16 and achieving the ideal waist-to-hip ratio (0.7) is encouraged. 5 9 However, the shape and projection of the buttocks should reflect the patient's ethnicity and desires. 3 Recent studies have further validated this approach, demonstrating improved aesthetic outcomes with minimal complications when proper technique is employed. 17

Indian females starve to achieve a subtle enhancement of contours in contrast to African Americans who aspires to have an overly projected and much wider buttock.

Involving patients in the decision-making process regarding fat volume and the area of maximum projection is crucial. The goal is to create an hourglass figure when viewed from the back and a curvy “S” shape from the side, aligning with the patient's ethnic characteristics and aesthetic preferences.

This study highlights the effectiveness of SSBA in achieving aesthetic improvements with minimal complications in the Indian population. Future research should involve larger, diverse patient samples, long-term follow-up, and objective assessments of patient satisfaction. Standardizing techniques, controlling variables, and exploring ethnic and demographic differences will improve result consistency and applicability.

## Conclusion

SSBA is a transformative procedure that, when performed by skilled surgeons, offers remarkable body reshaping results. With a strong foundation in gluteal anatomy, advanced technology, and strict adherence to safety guidelines, it ensures optimal patient outcomes. As body contouring continues to grow in demand, SSBA stands out as a safe and effective way to enhance physique.
